# Dietary Intakes and Exposures to Minerals and Trace Elements from Cereal-Based Mixtures: Potential Health Benefits and Risks for Adults

**DOI:** 10.3390/nu17172848

**Published:** 2025-08-31

**Authors:** Martina Mrázková, Daniela Sumczynski, Lenka Šenkárová, Richardos Nikolaos Salek

**Affiliations:** 1 Department of Food Analysis and Chemistry, Faculty of Technology, Tomas Bata University in Zlín, Vavrečkova 5669, 760 01 Zlín, Czech Republic; mmrazkova@utb.cz; 2 Department of Environmental Protection Engineering, Faculty of Technology, Tomas Bata University in Zlín, Vavrečkova 5669, 760 01 Zlín, Czech Republic; senkarova@utb.cz; 3 Department of Food Technology, Faculty of Technology, Tomas Bata University in Zlín, Vavrečkova 5669, 760 01 Zlín, Czech Republic; rsalek@utb.cz

**Keywords:** dietary intake, dietary exposure, cereal-based mixture, functional food, essential mineral, essential trace element, toxic trace element, ICP-MS, metal pollution index

## Abstract

Background: Foods containing nutraceuticals from the mineral element group are being developed to compensate for the problem of deficiency in billions of people around the world. This research focuses on essential elements of patented cereal-based mixtures to complement the deficiencies of these elements and, at the same time, assesses their safety in terms of toxic elements in the human diet. Methods: The mineral and trace element contents in the mixtures were determined using the ICP-MS method with a subsequent evaluation of the contributions of the mixtures to the essential and toxic reference values based on dietary intakes and exposures for adults at 60, 80 and 100 kg of adult body weight and a portion size of 50 g. The potential health risk was evaluated using a metal pollution index. Results: The concentrations of minerals and trace elements in the cereal-based mixtures analyzed were as follows: K (up to 4150 µg/g) ≥ P > Mg > Ca > Na > Fe > Zn > Mn > Cu > Al > Ba (up to 4.40 µg/g) > Sr (up to 480 ng/g) ≥ Ti ≥ Ni > Ce ≥ Co > As ≥ Cs > Ag ≥ Li > Se > Be > Cr > Tl > Pb ≥ Hg > Ho > Cd > Sn (up to 1.12 ng/g). The mixtures contribute significantly to the reference values for Mn, Cu, Zn, Fe, and P for adults. Individual dietary exposure values of toxic elements for adults weighing 60 kg decrease in this order: Al (10.1 µg/kg bw/day) > Ni (362 ng/kg bw/day) > As ≥ Pb > Ag > Hg > Cd > and Sn (0.93 ng/kg bw/day). Conclusions: In terms of Regulation (EU) No 1924/2006 of the European Parliament and of the Council on nutrition and health claims made on foods, the cereal-based mixtures could be labelled “source of” Mn, Cu, Zn, Fe, and P when their contributions to the reference values exceeded 15%; in addition, “low sodium/salt” or “very low sodium/salt” can be applied. The mixtures contribute insignificantly to the toxic reference values of Al, Sn, Hg, Cd, Ni, and Ag, and the exposure values of Pb for developmental neurotoxicity, nephrotoxicity, and cardiovascular effects were considered safe. Regarding the metal pollution index of mixtures, there is no concern for potential health effects. Cereal-based mixtures are suitable for use in the food industry as a potential source of beneficial micronutrients for the human diet, although bioaccessible studies should not be neglected.

## 1. Introduction

More than three billion people around the world are negatively affected by deficiencies in minerals and trace elements. Fe deficiency affects more than 25% of the global population [1,2], while the most vulnerable are children and pregnant women. The estimated prevalence of anaemia is 42% in pregnant women and 30% in nonpregnant women. In reality, 56 million pregnant women and 468 million nonpregnant women are affected by anaemia around the world [1,3]. Furthermore, vegans and vegetarians are at high risk of deficiency of Fe because they only consume Fe that is not haemified [4]. Symptoms of anaemia usually include fatigue, a weak immune system, and impaired brain function [5]. Additionally, more than half of nonpregnant women with deficiencies in Fe and Zn live in eastern Asia and the Pacific or southern Asia [6]. In more than a tenth of the population, the intake of Zn with meals is less than half the recommended dose, and chronic deficiencies in Zn significantly increase the risk of cancer, resulting in symptoms such as loss of smell or taste, hair loss, and diarrhoea [2,7]. Diets that offer insignificant Ca intake can also lead to nutritional deficiencies, such as osteoporosis in older adults and impaired physical growth [8]. Ca mineralises teeth and bones, especially during times of rapid growth; furthermore, it serves as a signalling molecule for heart, nerve, and muscle function [9]. In particular, populations in parts of Africa, Asia, and South America are at risk of low Ca intake [2]. In the USA, fewer than 10% of women over 50 years of age and men over 50 years of age met the recommended Ca intake [9]. What was more interesting was that close to 70% of the US population under 71 years of age, and about 80% over 71 years of age, consumed less than the required amount of Mg. Low Mg intake and its blood level are associated with metabolic syndrome, heart disease, type 2 diabetes, and osteoporosis [10].

It is evident that certain groups of consumers may be at risk of mineral deficiencies in the diet. Under this condition, subsequent supplementation under medical supervision and/or changing eating habits using raw materials with higher nutritional value is recommended. Today, consumers are demanding increasingly attractive, healthy, and tasty meals in which non-traditional cereals and pseudocereals, fruits, nuts, seeds, and even edible flowers as a component of meals can play a significant positive role in all the aspects mentioned. In addition, consumers believe that they positively influence not only the nutritional value of food but also directly improve their physical and mental well-being [11,12,13]. In the last decade, an increasing interest in nutraceuticals that form functional foods (for example, as breakfast) that combine proven physiological and nutritional benefits and medicinal values has been observed [12,14]. Essential minerals and trace elements (P, K, Ca, Mg, Zn, Mn, Cu, and Fe) play an important role in maintaining metabolic function, fluid balance, blood pressure regulation, nerve transmission, and immune system health [15].

Dry forms of edible flowers (for example, rose, lavender, hibiscus, mallow, red or blue cornflower, etc.) are considered to be a brilliant component of meals due to biologically active substances such as polyphenols, vitamin C, carotenoids, essential oils, minerals and trace elements, dietary fibres, with respect to high nutritional values and antioxidant activity status [11,13,16]. Edible flowers were traditionally consumed for their medicinal properties, while recently scientists have emphasised their nutritional [13]. Thus, the amounts of essential minerals and trace elements in the dry form of edible flowers are comparable or higher than those assessed for common fruit species, especially in terms of the amounts of P, K, Fe, Zn, Mn, and Cu [11,16]. In particular, wheat, corn, and rice are related to antioxidant or anticarcinogenic activities [12]. Although these grains are a necessary part of daily meals, they are devoid of essential micronutrients [17]. The facts mentioned inspire researchers to disseminate knowledge on the production and use of non-traditional pigmented grains (teff, millet, red wheat, kamut, black and red rice) or alternative cereals and pseudocereals (quinoa, buckwheat, amaranth) with a high mineral and trace element content, which could, for example, be part of cereal-based mixtures [17,18,19,20,21]. These non-traditional crops are more valuable in minerals and trace elements (i.e., P, K, Ca, Mg, and Mn) [12,15,19,20,21]. Hemp, flax seeds and nuts have been reported to be typical sources of proteins, oils (especially rich in ω-3 fatty acids), fibres, vitamins, and essential minerals (for example, P, K, Mg, Ca, Fe, Zn, Mn, and Cu) [12,22]. Hemp is newly grown for medicinal and food purposes, whereas some of its bioactive properties are attributed to phenolic compounds [22]. Almonds also provide biologically active substances, including phytosterols and phenolics; in addition to the higher content of essential elements, oilseeds and nuts are considered one of the food commodities that have the potential to accumulate toxic elements [23]. Thus, they are under the control of the European Commission [24]. Thousands of research studies investigated the nutritional value and health benefits of different types of fruits, juice and their waste by-products, etc. [25,26,27]. The interest of consumers in berry fruits has recently grown due to the higher content of essential minerals and trace elements such as Cu, Fe, I, K, Mg, and Mn [26]; for example, the main essential elements of goldenberries and barberries are K, P, Fe, Zn, Cu, Mg, Ca, and Mn [25,26,27].

Interest in healthy foods increases their consumption, which is related to the health benefits they provide, rather than their nutritional values [12]. These types of new foods could also be a source of both essential (P, K, Ca, Mg, Mn, Fe, Zn, and Cu) and toxic elements (Ni, Pb, Cd, As, Sn, Hg, and Al). The toxicity of some elements can cause negative changes in the body, such as neurotoxic changes. Intake of toxic elements can also contribute to the development of cancer and chronic kidney disease [28,29,30]. As a new type of healthy food, cereal-based mixtures consisting of non-traditional flakes, fruits, nuts, seeds, and edible flowers have already been patented under the trade name “Nutraceutical food mixtures” [18]. Taking this knowledge into account, this study aims to prepare cereal-based mixtures, which could be consumed most frequently as breakfast cereal mixtures; furthermore, they could potentially, when consumed regularly, address deficits in the intake of essential minerals and trace elements in the adult population or pregnant and lactating women. The promising hypothesis that the unique composition of cereal-based mixtures could significantly contribute to the daily intake of essential elements and their reference intake values has not been examined yet. However, since there is still a shortage of data on the minerals and trace elements of increasingly popular mixtures, not only toxic trace elements in foods need to be evaluated.

Following the above information about new types of patented cereal-based mixtures, this research on nutrition studies focuses mainly on essential and non-essential minerals and trace elements with the aim of supplementing the relevant deficiencies of these elements in the human diet and, at the same time, evaluating their safety in terms of the content of toxic trace elements.

In relation to cereal-based mixtures, the profile of minerals and trace elements, their daily intake, and exposures, including their metal pollution index, have not been reported in detail in the literature until now. The primary objective of our study was to investigate the content of Na, Mg, Al, P, K, Ca, Mn, Fe, Cu, Zn, Ba, Li, Be, Ti, Cr, Co, Ni, As, Se, Sr, Ag, Cd, Sn, Cs, Ce, Ho, Ta, Hg, Tl, Pb, and U in mixtures using the ICP-MS method. Additionally, this research estimates the contributions of cereal-based mixtures to the recommended dietary allowance (RDA), adequate intake (AI, when RDA is not defined), provisional tolerable weekly intake (PTWI), provisional tolerable monthly intake (PTMI), tolerable daily intake (TDI), and oral reference dose (RfD) for individual essential and toxic trace elements, based on the evaluation of daily intake (DI) and exposure (DE_bw_) for adults at 60, 80, and 100 kg of adult body weight. Furthermore, our evaluation included the metal pollution index (MPI) as a risk assessment value for human health. The details of these views are expected to be useful for the exploitation of cereal-based mixtures in the food industry because cereal-based mixtures could be a potential source of beneficial micronutrients for the human diet.

## 2. Materials and Methods

### 2.1. Raw Material and Production of Cereal-Based Mixtures

The grains with colored coating layers of *Oryza sativa* and *Chenopodium quinoa*, purchased on the market in Zlín (Czech Republic), were cooked for 25–30 min in an 80 °C water bath with a thermometer control. The flakes were then finalised with a Combi-Star flake roller set at a thickness of from 0.10 to 0.20 mm (Waldner Biotech, Lienz, Austria) and dried at 40 °C until the moisture content was less than 14% [31]. Regarding other flakes used in cereal-based mixtures, such as oat (*Avena sativa*), kamut (*Triticum turgidum* subsp. *turanicum*), red wheat (*Triticum aestivum* var. *milturum*), rye (*Secale cereale*), white teff (*Eragrostis tef*), and white quinoa flakes (*Chenopodium quinoa*), these were obtained in five packages of 300–500 g at a health food store in Zlín (Czech Republic).

Edible flowers in dried form of cornflower (*Centaurea cyanus*), rose (*Rosa centifolia*), lavender (*Lavandula angustifolia*), hibiscus (*Hibiscus sabdariffa*) and mallow (*Malva sylvestris* var. *mauritiana*) were supported in the amount of a package of from 100 to 300 g by Sonnentor and Oxalis (Čejkovice and Slušovice, respectively, Czech Republic).

Other ingredients such as dried or lyophilised barberries (*Berberis* spp.), goldberries (*Physalis peruviana*), strawberries (*Fragaria* spp.), apples (*Malus* spp.), raspberries (*Rubus* spp.), cherries (*Prunus avium*), and blueberries (*Vaccinium myrtillus*), as well as almonds (*Prunus dulcis*) and hemp seeds (*Cannabis sativa*) were purchased in a package of three bags of 50 to 200 g at the local market in Zlín, Czech Republic.

**Figure 1:**
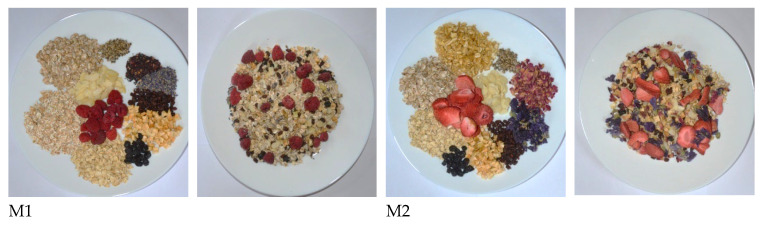

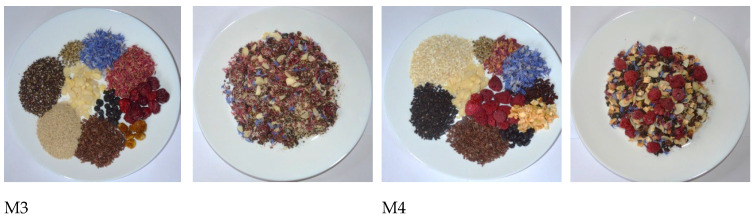
Novel non-traditional cereal-based mixtures prepared following the recipe presented in Table 1.

For the purpose of this study, four different cereal-based mixtures were prepared; gluten-containing mixtures were marked as M1 and M2, gluten-free mixtures were labelled as M3 and M4. A kilogram of each mixture contained 600 g of non-traditional flakes, and the remaining 400 g consisted of dried edible flowers, fruits, and nuts. Detailed compositions of the analysed mixtures are provided in Table 1 and Figure 1. All samples were kept in an air-conditioned laboratory without sunlight at a temperature of 23 ± 2 °C for no longer than one month prior to analysis.

### 2.2. Sample Preparation

The cereal-based mixtures were processed within 1 week after preparation by acid decomposition, applying a Milestone Ethos One microwave system (Sorisole, Italy) equipped with Teflon digestion vessels. First, to establish the mineral content, the mixtures were homogenised using a hand grinder. Seven millilitres of 67% ultrapure HNO_3_ and 1 mL of 30% ultrapure H_2_O_2_ were added to 0.20 g of homogenised sample. After closing the vessels, the digestion conditions were as follows: 1500 W for 15 min at 200 °C. Subsequently, the vessels were cooled to laboratory temperature, and the sample volume was diluted to 25 mL using 18.2 MΩ.cm of ultrapure water (Purelab Classic Elga system, LabWater, London, UK). The final samples were immediately analysed using ICP-MS (Thermo Scientific iCAP Qc, Thermo Scientific, Waltham, MA, USA).

### 2.3. Mineral and Trace Element Analysis

The determination of 31 elements was performed using an inductively coupled quadrupole plasma mass spectrometer iCAP Qc (ICP-MS, Thermo Scientific, Waltham, MA, USA) equipped with a collision cell (QCell) containing He for removing undesirable molecule ions by distinguishing their kinetic energy (CCT collision cell technology with kinetic energy discrimination mode). The detected isotopes were the following: ^7^Li, ^9^Be, ^23^Na, ^24^Mg, ^27^Al, ^31^P, ^39^K, ^40^Ca, ^48^Ti, ^52^Cr, ^55^Mn, ^57^Fe, ^59^Co, ^60^Ni, ^63^Cu, ^66^Zn, ^75^As, ^77^Se, ^88^Sr, ^107^Ag, ^111^Cd, ^118^Sn, ^133^Cs, ^137^Ba, ^140^Ce, ^165^Ho, ^181^Ta, ^202^Hg, ^205^Tl, ^208^Pb, and ^238^U. In terms of sensitivity and background signals, the instrument was tuned using solutions with Ba, Bi, Ce, Co, In, Li, and U containing 1 μg/L of each element in 2% HNO_3_ + 0.5% HCl; Ag, Al, Ba, Be, Bi, Ce, Co, Cs, Cu, Ga, Ho, In, Li, Mg, Mn, Ni, Rh, Sc, Sr, Ta, Tb, Tl, U, Y, and Zn containing 1 μg/L of each element in 2% HNO_3_ (Analytika, Prague, Czech Republic). ICP-MS was calibrated using external certified standard solutions to match concentration ranges of about 3–35 µg/L (Li, Be, Mg, Al, Mn, Co, Ni, Cu, Zn, Sr, Ag, Cs, Ba, Ce, Ho, Ta, Tl, and U) and calibration range curves of from 0.5 to 1.0 µg/L in the measurement of Na, P, K, Ca, Ti, Cr, Fe, As, Se, Cd, Sn, Hg, and Pb (Analytika, Prague, Czech Republic). A solution of Rh and In with a concentration of 5 µg/L was used as an internal standard (Analytika, Prague, Czech Republic). The precision of the method was verified against three certified reference materials (1568b rice flour released by the National Institute of Standards and Technology and purchased from Analytica Ltd., Prague, Czech Republic; and lichens and tea leaves provided by the Atomic Energy Agency, Vienna, Austria), with recovery of between 96.2 and 104% for all certified elements. These recoveries can be considered acceptable for the purpose of this research. For each element, the LOQ was calculated as 10 times the standard deviation of the signal from the prepared blank and analysed nine times [20].

### 2.4. Nutritional Intake of Essential Minerals and Trace Elements

Since no recommended daily intake of cereal grains or cereal mixtures for breakfast is defined, a daily serving size of 50 g was suggested for this research. A 50 g serving corresponds to one commercial package of cereal porridge, which is commonly available in food stores. Daily intake values (DIs) of essential minerals and trace elements of mixtures were calculated and evaluated with a comparison with the corresponding RDA or AI values as recommended by the National Academy of Medicine (NAM), known as the Institute of Medicine (IOM) until 2015 [32,33,34,35]. The individual level of dietary intake was determined for young adults (aged 19–30), pregnant and lactating women (aged between 19 and 30), middle-aged adults (aged 31–50), old-aged adults (aged 51–70), and seniors (aged over 70). The prescribed doses for the RDA and AI values for individual elements are declared in Table S1.

The daily intake of essential minerals and trace elements from the consumption of the mixtures was calculated using Equation (1).

DI = C × SS,(1)

where DI is the daily intake (mg, µg or ng per day), C is the mineral concentration of the mixture (mg, µg or ng/g), and SS is the serving portion of the mixture (50 g).

Contributions of the consumption of mixtures to the RDA or AI reference values for minerals and trace elements expressed as dietary intake estimate were calculated according to the following Equation (2):

DIE = (DI/RDA or AI) × 100,(2)

where DIE is the estimate of dietary intake (%) and DI is the daily intake (mg, µg or ng/g) RDA or AI (mg or µg/day, reference values defined for women and men in different stages of life, Table S1).

### 2.5. Evaluation of Diet Exposure to Toxic Trace Elements

Daily intakes (DI) of toxic elements from the consumption of the mixtures were calculated using Equation (1). Appropriate levels of daily exposure to the diet by body weight (DE_bw_, µg or ng/kg bw/day) for toxic trace elements were calculated for the mean human body weights of 60, 80, and 100 kg as follows (Equation (3)) [36].

DE_bw_ = DI/bw, (3)

The levels of exposure intake of elements Sn, Al, Hg, and Cd were evaluated and compared with the values of PTWI or PTMI; in the case of toxicity of Ni and As, the reference values of TDI were mentioned [37,38,39,40,41,42]. The level of Ag intake was compared to its RfD value [43]. The individual prescribed reference values are summarised in Table S1. Similarly, the appropriate contributions of the mixtures to the reference values of PTMI, PTWI, TDI and RfD for toxic trace elements were calculated according to Equation (4) and interpreted as the dietary intake estimates for toxic elements (DIE_TE_, %).

DIE_TE_ = (I/(RV × bw)) × 100, (4)

where DIE_TE_ is an estimate of the intake of toxic elements from the consumption of mixtures (%), I is the intake of trace element (µg per month, week or day), RV is defined as the reference value for toxic elements (µg/kg bw), and bw is body weight (kg).

Regarding the risk evaluation for Pb, the approach of margin of exposure (MoE) was used (Equation (5)), considering the respective individual BMDL values as follows: developmental neurotoxicity (BMDL of 0.5 μg/kg bw/day), nephrotoxicity (BMDL of 0.63 μg/kg bw/day), and cardiovascular effects (BMDL of 1.5 μg/kg bw/day), which are prescribed by [44].

MoE = BMDL/DE_bw_, (5)

### 2.6. Metal Pollution Index Analysis

To examine the overall concentrations of heavy metals in the mixtures analysed, the metal pollution index (MPI) was calculated as the geometric mean of the concentrations of heavy metals in the samples (µg/g) [36]. The MPI index was computed using the following Equation (6), where C_1_, C_2_ …and C_n_ are the concentrations of an individual metal in the cereal-based mixtures.

MPI = (C_1_ × C_2_ × …C_n_)^1/n^, (6)

### 2.7. Statistical Analysis

All analyses were repeated nine times, and the results were expressed as mean ± standard deviation on the basis of dry weight. The use of non-parametric tests was rejected because all results were accepted for the normal distribution (Shapiro–Wilk test; significance level 0.05; Minitab^®^ 16 software; Minitab Ltd.; Coventry, UK). Thus, the obtained results were processed using a one-way variation analysis (ANOVA, TriloByte Statistical software, QC expert 3.3. Pardubice, Czech Republic). Subsequently, the Student’s *t*-test was used to determine differences in the mean value, and the level of significance was set to 5% (*p* < 0.05).

## 3. Results and Discussion

### 3.1. Mineral and Trace Element Content of Cereal-Based Mixtures

The contents of 31 elements in the samples were determined using ICP-MS. The results values measured in µg or ng/g values on dry weight are summarised in Table 2. It is proven that the concentrations of mineral elements in plant raw materials are affected by many factors, such as the amount of diverse elements in contaminated soil and the statement in polluted air, rainfall during growth, fertilisation, the level of ripeness at the time of harvest and by technological treatments, including steaming, drying, and flaking [45]. There are scant data on the composition of the mineral and trace elements of the mixture in the available literature, and, therefore, to compare the results of our study, we also looked for values of the mineral and trace element content in the individual raw materials that we used to prepare the mixtures.

As can be seen in Table 2, minerals and trace elements were assessed in the following order: K (up to 4150 µg/g) ≥ P > Mg > Ca > Na > Fe > Zn > Mn > Cu > Al > Ba (up to 4.40 µg/g) > Sr (up to 480 ng/g) ≥ Ti ≥ Ni > Ce ≥ Co > As ≥ Cs > Ag ≥ Li > Se > Be > Cr > Tl > Hg ≥ Pb > Ho > Cd > Sn (up to 1.12 ng/g). Because of the unique heterogeneous composition of cereal-based mixtures, it is very difficult to discuss the measured data as such in their values. Our results show that the mixtures contain the highest concentrations of K and P, followed by Ca and Mg. These elements are abundantly present in all plant-based ingredients used in mixtures. Cereals, pseudocereals, almonds, hemp seeds, goldenberries and edible flowers are especially rich sources of K, P, and Ca [13,15,21,23,25]. Specifically, P is stored in cereals and seeds in the form of phytates, which show an antinutritional effect on the absorption of Ca, Fe, K, Mg, Mn, and Zn [46,47]. Mg is mainly contained in almonds, hemp seeds, and cereal grains, which are part of mixtures [15,21,23]. In contrast, cereals are poor in Mn, whereas a higher amount of Mn was observed in hemp seeds and edible flowers [16,21]. To prevent high blood pressure, it is essential to obtain a low Na concentration in foods that meet the mixture. Recent studies have shown that Na sources are strawberries, goldenberries, or edible flowers, especially hibiscus [16,25]. Concerning Fe, Zn, Cu, and Co contents in cereal-based mixtures, their higher values may be due to the presence of non-traditional flakes with a higher proportion of coating layers, hemp seeds, as well as the presence of blueberries, barberries, raspberries, and edible flowers in the recipe of the mixtures [11,21,22,26,27]. In more detail, wheat grains contain up to three times more Fe and twice as much Zn as rice grains [17]. In contrast, wheat grains contain one and a half times less Fe and half the amount of Zn than red and black quinoa grains [47,48].

In our study, toxic elements such as Hg, Pb, Sn, Al, Cd, As, and Ni were monitored. The mean value of the Cd content in cereals consumed in the EU varies from 0.4 to 6.0 µg/100 g, whereas the maximum Cd value in cereals has been established at 100 µg/kg [24]. As can be seen from the results, the Cd concentrations were assessed in lower concentrations than was set by this limit. Similarly, Com. Reg. 915/2023 [24] established the maximum lead level for cereals of about 200 µg/kg. As can be seen from our results (Table 2), the Pb content was below this limit. The maximum concentration levels of the remaining toxic elements, including Sn, Hg, and Ni, have not been established yet. Since the bioavailability of Ni from breakfast cereals is 99.6%, it is important to monitor its content [49] as well as its amount in other raw materials (e.g., hemp seeds) [12].

To our best knowledge, the amounts of trace elements (such as Ba, Li, Be, Ti, Cr, Se, Sr, Ag, Cs, Ce, Ho, Ta, Tl, and U) in cereal-based mixtures have not been well documented. All of the trace elements mentioned above are commonly found in plant matrices, and many of them are also embedded in the geological origin (especially Li and Ti). None of the trace element concentrations measured in mixtures exceed the commonly published ranges of their concentrations in plant foods [50].

### 3.2. The Results of Daily Intake of Essential Minerals and Trace Elements

**Figure 2:**
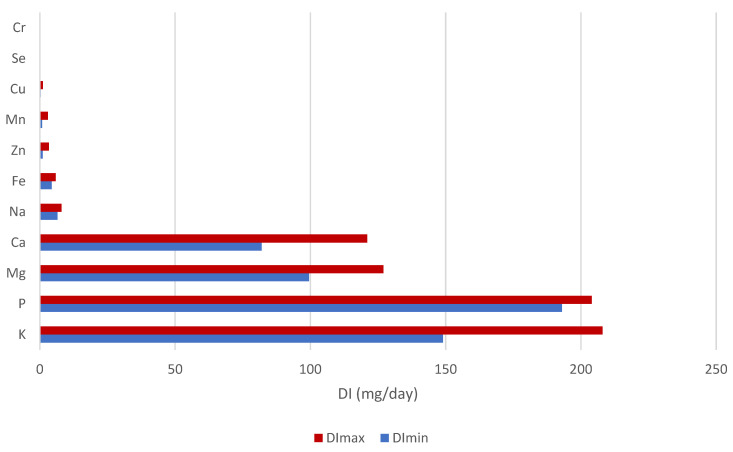
Minimal and maximum daily intake values (mg/g) of essential minerals and trace elements in the diet of 50 g cereal-based mixtures.

The maximum and minimum daily intake (DI) values of essential minerals and trace elements when consuming a 50 g portion of cereal-based mixtures simulated as one portion of breakfast are presented in Figure 2. It is evident that the highest DI values were obtained in the case of K and P intake, followed by daily intakes for Mg and Ca, when the DI_max_ value may reach up to 208 and 204 mg per day, respectively. It is generally known that K and P are present in natural plant foods, in particular fruits, whole grains, and legumes. According to the EU data sheet, the average K and P intake ranged between 2463 and 3991 and 1000 and 1767 mg/day in adults (≥18 years of age), respectively [51].

The EFSA Panel on Dietetic products, Nutrition and Allergies (NDA) noted that zero Mg balance can occur at a Mg intake of 165 mg/day and pointed to an inverse relationship between Mg intake and the risk of type 2 diabetes [51]. Regarding the consumption of 50 g portions of mixtures, the DI values for Mg range from 99.5 to 127 mg/day, which could significantly positively influence the Mg intake balance in the adult diet, as the main food groups contributing to Mg intake are cereals [51]. Dairy products are the main sources of Ca in human nutrition [51], definitely. Despite this fact, if we study the raw material composition of mixtures, then nuts can be considered a main source of Ca, followed by pseudocereals. For example, a serving (28.4 g) of almonds provides 76 mg of Ca, 136 mg of P, and 208 mg of K, which represent 8, 14, and 6% of the daily value, respectively [23]. Ca, Mg, and Fe are elements that have been proven to be deficient in the diets of celiac people [48]. Therefore, the incorporation of quinoa and almonds as parts of cereal-based mixtures into the diet of celiac patients should help alleviate at least a part of the deficit in intake in this sector of the population and also increase the consumption of whole grains.

The EFSA Panel considered that the Na intake of 2000 mg/day represents a level for which there is significant confidence in a reduced risk of CVD (cardiovascular disease), and this suggested value is safe and adequate for the general adult population [51]. The daily intake of Na provided by the consumption of the mixture is very low (only 8 mg/day); therefore, its consumption may contribute to a lower intake of Na, which exceeds the adequate and safe intakes established for Na in the EU [51]. The DI values of Fe from 50 g of mixtures range from 4.38 to 5.85 mg/day (Figure 2).

The new types of cereal-based mixtures could account for from one-quarter to one-half of the average Fe intake, which is between 9.4 and 17.9 mg per day in the diet in the EU [52]. Plant ingredients that contain relatively high amounts of Fe include nuts, cereals, and seeds [52], which are constituents of mixtures. A 50 g portion of quinoa with colored coating layers can be a source of up to 7.45 mg Fe per day, compared to oat grains, where it can be 4.7 mg Fe per day. In the case of flax and hemp seeds, we can supplement our daily Fe intake by up to 13 mg [12].

Instead of meat and milk-based products, whole-grain cereal products are known to be rich sources of Zn in the diet [51]. When consuming a 50 g portion of the cereal-based mixture, the DI values range from 1.08 to 3.35 mg Zn per day. Taking a 50 g serving of oats or quinoa, a daily zinc intake of around 2.93 mg can be achieved, while consuming the same serving of oilseeds provides one third more [12]. EFSA states that the average daily intake of Zn in the diet of an adult is at least 8.0 mg [51].

Several balance studies have been carried out to demonstrate adaptability to changes in Mn intake. The balance metabolism of Mn may be maintained with an intake of less than 2.5 mg/day and may be influenced by the entire diet. No scientific data on Mn intake and health outcomes were identified for the setting of CVDs [51]. In Europe, the estimated average Mn intake for adults ranges between 2 and 6 mg/day, usually somewhere around 3 mg/day [53]. As can be seen from our results, the DI value for Mn is in a fairly wide range (from 0.87 to 2.99 mg/day) (Figure 2). Specifically, the intake value of 3 mg Mn per day can be achieved by consuming just one serving of a gluten-free mixture labelled M4. The Mn DI value from the consumption of 50 g of cereal or oil seeds may reach approximately 2.65 mg/day [12].

The trace element whose intake from the consumption of mixtures reaches values in mg per day (concretely up to 1.14 mg) is Cu. Regarding the composition of mixtures, the rich dietary sources of Cu are nuts, seeds, edible flowers, and cereals [11,12,54]. According to the EFSA Panel, the average Cu intake is between 1.15 and 2.07 mg/day, whereas the average intakes were in most cases slightly higher in men than in women [54]. Relevantly, the EFSA Scientific Committee has noted that Cu retention should not occur within an intake of 5 mg/day and suggested an ADI (acceptable daily intake) of 0.07 mg/kg bw [55]. Considering our measurements, we agree with the claim already underlined by Gu [12] that the concentrations and consequently daily intake values of Cu from consumption of 50 g of oats, quinoa, hemp and flax seeds, ranging from 390 to 1740 µg/day, are quite high and comparable to our results.

Cereal-based mixtures provide daily Se and Cr intake in tenths of micrograms, from 0.4 to 0.9 and from 0.1 to 0.4 µg/day, respectively (Figure 2). The Se content of plants generally depends on the amount of Se in the soil, as well as its geochemical characteristics, such as the uptake of Se depending on soil pH, redox potential, and water content. For example, nuts are known as Se accumulators, while wheat, rye, oats, and barley are non-accumulators [56]. With regard to these generally known facts, the source of Se in mixtures will be mainly nuts and seeds, and only then the grain portion. In the EU, the average Se intake from food consumption ranges from 31 to 65.6 µg/day in adults [56]. It is evident that mixtures represent only trace amounts of Se daily intake. Currently, the median dietary Cr intake in the population in the EU is in the range of 57.3 and 83.8 µg/day in adults [57,58]. Given that the main contributors to daily Cr intake are milk and dairy products, bread, rolls, and chocolate, a lower intake of Cr can be expected from cereal-based mixtures.

### 3.3. Evaluation of Dietary Intake Estimates to Reference Values for Essential Elements

**Figure 3:**
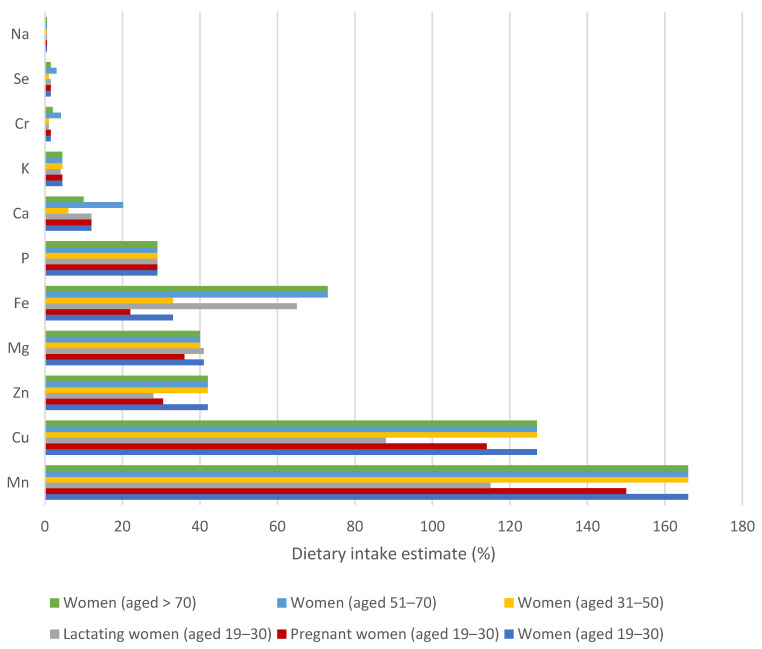
Dietary intake estimate (%) values of essential minerals and trace elements in the diet of 50 g of cereal-based mixtures for women in a defined stage of life.

**Figure 4:**
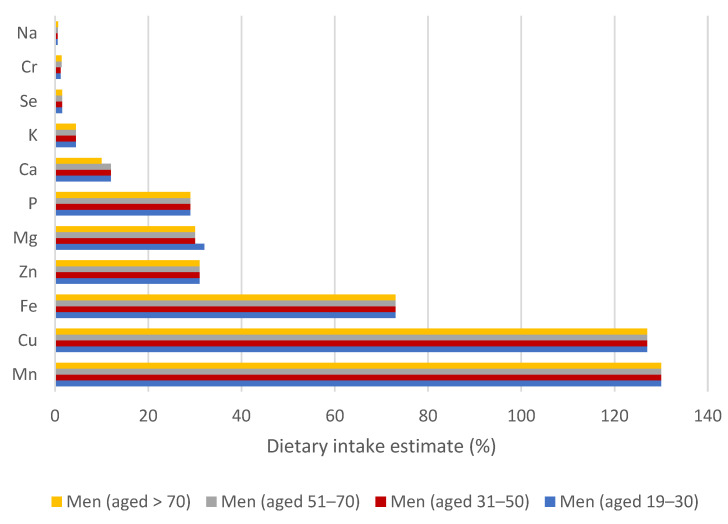
Dietary intake estimate (%) values of essential minerals and trace elements in the diet of 50 g of cereal-based mixtures for men in a defined stage of life.

Figure 3 and Figure 4 show the results of the contribution of cereal-based mixtures to RDA (recommended dietary allowance) or AI (adequate intake) reference values for essential elements for women and men, and these are presented as DIE (dietary intake estimate) in percentages. The results were calculated by connecting the ingestion of 50 g of the mixture per day for an adult. The results show that greater differences in DIE values were achieved in individual categories for women (Figure 3), compared to men (Figure 4). This is certainly due to the requirements for RDA or AI values, especially for lactating and pregnant women.

The highest DIE values for all age categories of women and men were obtained for the Mn intake, and all were above 100%. The lowest contribution of the cereal-based mixture to the AI value of Mn (115%) was evaluated in the category of lactating women, followed by pregnant women (150%) (Figure 3). Regarding all age categories of men, the contribution of the mixture to the AI value of Mn was uniform at 130% (Figure 4). Mn is a necessary element for blood formation and clotting, reduction of inflammation. In addition, it is a part of metalloenzymes such as superoxide dismutase, arginase, and pyruvate carboxylase, where it participates in cholesterol, lipid, amino acid, and glucose metabolism [16,53]. In the EU, the concentration of Mn in the common diet is relatively high; therefore, its deficiency does not occur. Furthermore, specific Mn deficiency disease has not been documented in humans. For example, 100 g of fresh rosehip can contribute more than 100% of Mn to the AI value [27].

Cu acts as a donor and acceptor of electrons in a biochemical reaction similar to that of Fe and is a component of the catalytic centre in enzymes, especially those involved in neurotransmitter synthesis [54], and is necessary for the proper growth and development of connective tissue, brain, and heart [16]. Regarding the wide range of enzymes containing Cu as a cofactor, the symptoms of insufficiency are rare or diverse: include normocytic and hypochromic anaemia, hypercholesterolemia, hypopigmentation of the skin and hair, leukopenia, neutropenia, myelodysplasia and, in most patients, neurological findings, most commonly due to neuromyelopathy [2,54]. Furthermore, Cu deficiency is associated with alterations in immune function and bone function; however, all these symptoms occur in other diseases, making it very difficult to distinguish Cu deficiency from the phenotype [54]. As the results reflect, the contribution to the RDA value for Cu stood out for men and women at all stages of life because the consumption of 100 g of cereal-based mixture would exceed the DIE value of 120%. However, for pregnant and lactating women, the estimated value of dietary intake is lower, at 114 and 88%, respectively, due to the requirement for a higher RDA value for Cu intake. For comparison, ingestion of 100 g of fruit per day for an adult represents only a slight contribution to the RDA of Cu, that is, only 0.9% [26]. The study provided by Sumczynski [59] concluded that a 100 g portion of wheat flakes strongly contributed to the RDA of Cu for all age groups, with the lowest contribution to the RDA of 48% and 69% for lactating and pregnant women, respectively.

With respect to Fe, the DIE values for the consumption of mixtures were 73% for all categories of men and women over 51 years of age (Figure 3 and Figure 4). For lactating women, the DIE value for Fe intake is 65%; for the category of women 19–50 years, it is 33%; finally, the lowest value was calculated for pregnant women (DIE = 22%), where the low intake value is given by the requirement of a high reference value for Fe intake (concretely 27 mg/day). If we compare the results with the consumption of a 50 g serving of hemp seeds and fresh fruit pulp by adults, these contribute to the reference intake value of Fe by 25 and 5%, respectively [22,26]. Compared to the consumption of 50 g of non-traditional cereal flakes, men of all ages and women aged from 51 to over 70 could obtain up to 8% of the RDA of Fe; contributions for women aged 19–50 decrease to 4%, for pregnant women to 3% and, ultimately, for lactating women, it reaches 7% [59]. Therefore, 50 g of the mixture could help to combat Fe deficiency in the human population [1,3]. Fe performs a central role in metabolic pathways that involve oxygen transport (haemoglobin), short-term oxygen storage (myoglobin), hem enzymes involved in electron transfer (cytochromes a, b, and c) and oxidase activities (cytochrome P450, peroxidases), and is important for blood formation, as well as oxidative metabolism and cell growth [3,16,26,52]. Many Fe deficiencies have traditionally been attributed to glossitis, koilonychia, soft nails, mood changes, cheilitis, muscle weakness, and impaired immunity. A low Fe content in the human body is associated with inefficient energy metabolism, altered lactate and glucose utilisation, impaired collagen synthesis, and osteoporosis, which may be due to impaired vitamin D hydroxylation [5,51,52].

Discussing Zn, significant differences were found between individual categories of women (Figure 3). The highest DIE value (42%) was calculated for all age categories of women from 19 years to older people over 70 years of age. Both EFSA and NAM considered additional Zn intake requirements during the first three trimesters of pregnancy (for the fetus, placenta and other tissues) and for lactating women (due to losses of Zn with human milk) [60]; therefore, the contributions of cereal-based mixtures to RDA values of Zn are lower, concretely, 31 and 28%, respectively. Regarding the category of men, the contribution of mixtures to the RDA value for Zn is always 31%. For example, regular consumption of a 50 g portion of hemp seeds and fresh fruits can contribute 50 and 4.5% to the recommended dietary allowance of Zn, respectively [22,26]. In contrast, non-traditional flakes were found to be an insignificant source of Zn with a value of DIE of 6% for women of all age categories [59]. Zn is known to support wound healing, immune function, and DNA synthesis, and its absence has been associated with depression and cognitive impairment in adults. It seems that the immune-boosting effect is partially responsible for reducing infection and inflammation-related burdens. Zn deficiency may also affect pregnancy outcomes by contributing to complications during pregnancy and childbirth, and it is important for milk secretion and fetal development [2].

The consumption of 50 g of cereal-based mixtures contributes significantly to the RDA value of Mg for all age groups included in this study. Non-traditional mixtures provide 40% of the RDA value of Mg for all women except pregnant women, where the contribution to the RDA value is 36% (Figure 3). Considering that pregnancy requires only a slight increase in Mg requirement, which is probably covered by adaptive physiological mechanisms, it is not important to increase the RDA value for Mg [61]. Regarding all groups of men (Figure 4), the Mg intake represented 30–32% of the RDA. It is generally known that Mg is a cofactor for hundreds of enzyme reactions acting as a catalytic component for the reaction of adenosine diphosphate (ADP) to adenosine triphosphate (ATP), making Mg essential for the nucleic acid synthesis of proteins and carbohydrates, as well as for specific actions in the neuromuscular or cardiovascular system [16,26,51]. Mg deficiency occurs only occasionally and can have many different causes, including renal and gastrointestinal dysfunctions, hypocalcaemia (associated with falls and fractures), and hypokalaemia, leading to cardiac and neurological symptoms [2,61]. Individuals may experience cramps, tremors, muscle weakness, and fatigue when Mg levels fall below the biochemical range [2].

The contribution of the consumption of cereal-based mixtures to the RDA value of P is relatively high, namely, 29%, for each category examined (Figure 3 and Figure 4), which is approximately three times higher than when consuming wheat flakes [59]. With regard to the raw material composition of cereal-based mixtures, the consumption of 30 g of hemp seeds contributed up to 50% to the RDA of P [22]. Since approximately 50% of P is bound in the coating layers in the form of phytic acid, cereal crops with these brans show a higher content of P; critically, the antinutrient effect of phytic acid associated with the affinity for the absorption of Fe, Zn, and Ca must be taken into account [59]. In context, hypophosphatemia occurs only rarely due to inadequate dietary P intake and is generally due to metabolic disorders; however, the P content in the balanced diet for adults appears sufficient [51,59]. In contrast, an excessively high dietary phosphate intake can lead to diseases such as type 2 diabetes [2]. P is used for various cellular processes, including cellular energy transport with ATP, involving intracellular signal transduction, bone stiffening, cell membrane formation, and nucleic acid synthesis [2].

It seems that cereal-based mixtures are an insignificant source of Ca [62] when consumed in a 50 g portion. With the exception of the category of women aged from 51 to 70 years, for other categories included in our study, the contributions to the reference values for Ca are lower than 15%; for the category of women aged 31–50 years, it is even only 6% (Figure 3 and Figure 4). Nevertheless, as with mixtures, wheat flakes themselves are an inefficient Ca source, as they provide less than 2% of the RDA for men and women [20]. Regarding the human body, approximately 99% of Ca is incorporated in bones and teeth, where it performs the function of structural support and serves as a reservoir to maintain the extracellular form of Ca levels. It is essential for muscle contraction, nerve signalling, and blood clotting. Ca deficiency is common and can activate poor calcification and changes in the dentition (osteoporosis), muscle cramps, and arrhythmia. In pregnant women, there is an increased risk of preeclampsia and related complications associated with inadequate Ca and vitamin D intake [2,16,51]. In contrast, older people are sensitive to excess Ca:Mg intake due to deteriorated renal function [2,3].

As the results reflect, when the AI for K is 4700 mg/day, then a 50 g portion of the cereal-based mixture can contribute 5% to its reference value (Figure 3 and Figure 4). Taking into account evidence that the total body K content decreased in lactating women, a conservative approach was taken into consideration, and the amount of K needed to compensate for K losses through breast milk is added to AI for adults [51]. In lactating women, the AI value is higher, and, therefore, the contribution of the mixture to its value is lower, namely, 4%. K is a predominant osmotically active mineral within the human body that controls the acid-base balance by distributing water within and outside cells and establishes a membrane potential in cells that supports electrical activity in nerve and muscle fibres [2,51]. K deficiency, which presents as hypokalaemia, is usually caused by increased K losses (e.g., due to vomiting, diarrhoea, or excessive renal losses) or intracellular K changes. Hypokalemia due to a lack of K in the diet does not practically occur [51].

Consumption of 50 g of non-traditional mixtures does not significantly contribute to the AI value of Cr and Na, nor to the RDA value of Se (Figure 3 and Figure 4), with all contributions to the reference values for these elements being below 4%. Cr is reported to be an essential element significant for the activity of insulin in the regulation of carbohydrate, lipid, and protein metabolism [51]. Although Cr is important for these metabolic pathways, excessive intake could result in liver and kidney damage, skin diseases, and can even encourage many types of tumours [2]. Se has the ability to bind to toxic trace elements (e.g., Hg and Cd) and consequently contributes to a mitigation of various pathological effects. In addition, it is essential for the expression and function of selenoproteins, and deficiency can be related to Keshan and Kashin–Beck diseases, oxidative stress and inflammatory effects, diabetes, hepatopathies, infections, and an increased risk of certain tumours [2,56]. It is essential in the diet to obtain less than 0.7% of the AI value of Na to prevent hypertension for men and women of all age groups, as well as pregnant and lactating women. Na exists as a dominant electrolyte cation in the extracellular fluid of body cells and participates in the regulation of the distribution of water in the human body and generation through interactions with K of transmembrane electrochemical potentials [3,16].

The mineral and trace element content in cereal-based mixtures with edible flowers is nutritionally interesting. Taking into account the reference values for essential elements established by Regulation (EU) No 1169/2011 of the European Parliament and of the Council on the provision of food information to consumers [62], non-traditional cereal-based mixtures could be considered a significant source of Mn, Cu, Zn, Fe, and P because they reach a minimum of 15% of the reference value for the nutrient supplied by a 100 g portion. According to Regulation (EC) No 1924/2006 of the European Parliament and of the Council on nutrition and health claims made on foods [63], mixtures can be labelled “source of” Mn, Cu, Zn, Fe, and P because the mixtures contain at least a significant amount as defined in Regulation [62]; furthermore “low sodium/salt” or “very low sodium/salt” can be applied because the mixtures contain no more than 0.12 g and 0.04 g of Na, respectively, per 100 g of product. In this view, the presentation of nutritional claims could be partially confusing for the customer due to the linking to the presence of these compounds in 100 g of seeds without reflecting the serving size. Furthermore, it should be critically noted that the concentration of a nutrient in a food sample is not a good indication of the amounts of essential nutrients or toxic elements ingested. However, in order to acquire the real nutritional data or obtain accurate information on the risk of consuming cereal-based mixtures, bioaccessible studies are required in the future.

### 3.4. The Results of Dietary Exposures and Dietary Intake Estimates to Toxic Trace Elements

Regarding raw materials of plant origin, these can accumulate toxic trace elements (Ag, Al, As, Ni, Cd, Pb, Hg, and Sn) from the earth’s subsoil, irrigation water, and atmospheric dust. Moreover, fertilisers can significantly influence the uptake, especially of Cd, Pb, Hg, and As [50]. In addition to that, As, Cd, Hg, and Pb are listed in components of the substance priority list created by the Agency for Toxic Substances and Disease Registry [64]. Thus, not only the contamination of raw materials as components of mixtures should be of public interest, but also the contributions of mixtures to dietary exposure to toxic elements. Eight toxic trace elements were examined in four cereal-based mixtures. Table 3 summarises the levels of estimated dietary exposures by body weight (DE_bw_) from the consumption of 50 g of mixtures. Individual DE_60_ values for toxic trace elements of the cereal-based mixtures decrease in this order: Al (10.1 µg/kg bw/day) > Ni (362 ng/kg bw/day) > As ≥ Pb > Ag > Hg > Cd > and Sn (0.93 ng/kg bw/day).

In terms of Al, its highest diet exposure values were observed, concretely from 10.1 µg/kg per day for a person weighing 60 kg to 6.05 µg/kg per day for participants weighing 100 kg. As cereal crops and their products were evaluated as the main contributors to dietary exposure to Al, followed by fruits and vegetables [65], it can be expected that mixtures are a source of this element. Miscellaneous results were published in the study by Nekvapil [66], where the DE_60_ values of the consumption of 50 g of non-cereal flours were in the range from 4.4 to 22.9 µg/kg per day, when the highest value was found for the consumption of flax seeds and the lowest for grape seed flour. In the EU, the mean dietary exposures from food consumption represent values ranging from 0.2 to 1.5 mg/kg of body weight per week in a 60 kg adult [65].

As far as Sn is concerned, the main dietary source is most often an acidic type of food packaged in partially lacquered or unlacquered plated cans [37]. Due to the nature of mixtures, it is not expected that these will be packaged in plated cans and, therefore, should not be a significant source of Sn. The highest value of daily Sn intake from consumption of 50 g of mixture reaches 56 ng/day, which corresponds only to the dietary exposure of 0.93 ng/kg/day for an adult weighing 60 kg (Table 3).

In the EU, DE values for Hg from food consumption for adults were reported to vary for the mean between 0.07 and 0.43 µg/kg bw/day with a median of 0.16 µg/kg bw/day; furthermore, values ranging between 0.05 and 0.36 µg/kg bw/day were observed for elderly people [67]. It is obvious that the DE values from consumption of cereal-based mixtures are an order of magnitude lower, in nanograms. If we compare our measured data with other research, by consuming 50 g of wheat flakes, an estimated DE_60_ of only 6.0 ng/kg bw was found [59], whereas, from the consumption of mixtures, it was more than twice as much.

Regarding exposure to Cd, the main categories of foods that contribute to daily intake include cereal grains and vegetables [68]. Although the cereal portion makes up the majority of mixtures, as can be seen in our study, the value of DE_60_ is low, namely 12.9 ng/kg bw/day (Table 3). It should emphasise that other foods consumed per day can also contribute to exposure to Cd instead of cereal-based mixtures. For comparison, the DE_60_ value for Cd from food consumption in the US population was defined around 0.32 µg/kg bw/day; in most countries, the average DE_60_ value of Cd from foods is mainly lower (in the range of from 0.1 to 0.4 µg/kg bw/day) [69]. Regarding the EU databases, the mean dietary exposure values of Cd for adults were estimated to be in the range of 0.21 and 0.32 µg/kg bw/day [68].

As Ni is ranked among toxic trace elements whose highest daily intake is associated with the consumption of cereal products, its exposure to the consumption of mixtures was examined [70]. As Table 3 shows, a 50 g portion of the tested samples responds to a daily Ni exposure of 362 ng/kg bw/day for an adult weighing 60 kg. Regarding similar research, the highest DE_60_ value of 3690 ng/kg bw/day was estimated from the consumption of 50 g of milk thistle flour, where this sample was recognised as the most substantial contributor from the noncereal flours analysed [66]. The EFSA CONTAM Panel (Panel on Contaminants in the Food Chain) decided to establish a tolerable exposure of 13 μg Ni/kg bw/day, whereas chronic levels of Ni dietary exposure for the adult population in European countries were between 1.83 and 4.19 μg Ni/kg bw/day; furthermore, dietary exposures, specifically for pregnant and lactating women, were within the range of exposure estimates for the adult population [70].

Upon examination of cereal-based mixtures, the DE_60_ value of As of 50 g of portion consumption is 26.3 ng/kg bw/day, which is the same exposure as for the intake of Pb, and very close to the exposure calculated for the Ag intake. EFSA posted the DE_60_ values to As among adults ranging from 0.09 to 0.38 μg/kg bw/day [71]; notwithstanding, the NAM indicates that the exposure to As for all age groups is slightly higher, concretely from 0.50 to 0.81 µg/kg bw/day [34]. It is evident that the value of DE_60_ in As intake is below the intakes presented by NAM and EFSA. Compared to a similar study, the DE_60_ value measured for cereal-based mixtures is 3–10 times lower when the consumption of 50 g of cereal grains corresponded to exposures to As of from 86.5 to 259 ng/kg bw/day [72].

Humans are exposed to small amounts of Ag from dietary sources. Oral intake of Ag from a typical diet has been estimated to range from 27 to 88 µg/day, which corresponds to the DE_60_ value of approximately from 0.5 to 1.47 µg/kg bw/day [43]. Until now, the Joint FAO/WHO Expert Committee on Food Additives has not yet prepared any reference values; however, according to the European Medicines Agency guideline, the allowed oral Ag intake was established at 167 μg/day, which corresponds to the DE_60_ value of approximately 2.8 μg/kg bw/day [73]. Additionally, the US EPA suggested applying a reference oral dose of 5 µg of Ag per kg bw [43]. Regarding the daily consumption of 50 g of cereal-based mixtures, the value of DE_60_ is five times higher than in the case of the consumption of non-cereal flours [66].

**Figure 5:**
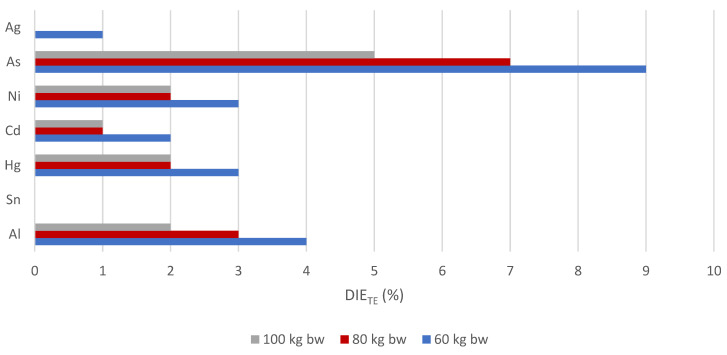
Dietary intake estimate (%) values of toxic trace elements in the diet of 50 g of cereal-based mixtures based on defined body weight.

With regard to toxic exposures to diet, it can be observed that cereal-based mixtures contribute insignificantly to the PTWI of Al, Sn, and Hg, to the PTMI of Cd, to the TDI of Ni and As, and finally to the RfD of Ag (Figure 5). Dietary intake estimates for toxic elements (DIE_TE_, %) were calculated using the individual prescribed reference values (Table S1) according to Equation (4).

Regarding the highest value of individual contributions, the estimated contribution of mixtures to the TDI value of As has not exceeded 9% for an adult weighing 60 kg. It should be emphasised that all remaining contributions of mixtures to toxic dietary exposure values for Ag, Ni, Cd, and Al are less than 4%, and, in the case of Sn, it is even below 0.1%. It postulates that all injurious trace elements are present in amounts well below the safe limit (Figure 5). When comparing data, milk thistle seed flour was found as an important contributor to the toxic reference values of Ni (57%), Cd (26%) and Hg (20%), where the results were presented in a 100 g serving size [66].

Long-term exposure to As causes cancers of the skin, lung, liver, bladder, and kidney; epigenetic modifications, oxidative stress, chromosomal aberrations with DNA damage have been proposed as carcinogenic modes of this element [29,71]. Regarding the second most exposed element, after absorption, Al is distributed to tissues and accumulates particularly in bone, can enter the brain and reach the placenta and fetus, and shows neurotoxicity in patients undergoing dialysis. Al has been suggested to be implicated in the aetiology of Alzheimer’s disease, but, based on the available scientific data, the Panel on Food Additives, Flavorings, Processing Aids and Food Contact Materials (AFC) does not consider exposure to Al via food to constitute a risk for developing this disease [65,74]. Exposure to Cd is related to liver dysfunction, cardiovascular disease, osteoporosis, and chronic kidney disease (CKD). When cereal grains can absorb Cd easily from the soil, this food commodity must meet the toxic limits for Cd applicable in the EU [24,30].

**Figure 6:**
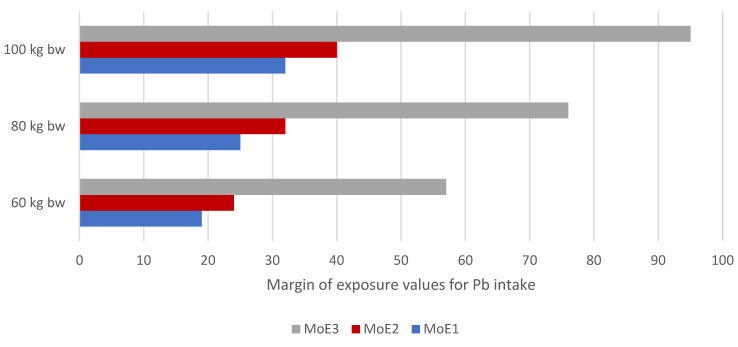
Margins of exposure values (MoE) for Pb intake in the diet of 50 g of cereal-based mixtures based on defined body weight.

Due to the chronic toxicity of Pb even at low concentrations, the allowed concentration limits in fruits and cereals were established [24]. This neurotoxin accumulates not only in bones but also in soft tissues. Pb toxicity is associated with chronic kidney and eye diseases, is dangerous to the nervous system, and causes brain disorders, especially in children [44,75]. Concerning the DE_60_ value of Pb, the consumption of 50 g of non-traditional cereal-based mixtures can possess 26.3 ng/kg bw/day (Table 3). When comparing the data, a 50 g portion of wheat flakes was associated with a daily intake of about 1.7–2.9 µg of Pb per day, with a range of DE_60_ values from 30 to 48 ng/kg bw/day [59]. The mean exposure to Pb in the diet for adults (individual estimates by countries of the EU) ranges from 0.36 to 1.24 μg/kg bw/day [76]. The margin of exposure approach, including BMDL values for neurotoxicity, nephrotoxicity, and cardiovascular effects, was suggested by the EFSA to characterise the health risk of Pb intake [44]. Individual MoE values obtained for current exposures from mixtures to Pb were more significant than 1 [77], which is evaluated as a safety margin without health concern (Figure 6). Based on limited studies comprising health risks using BMDL limits for Pb, to illustrate this point, the MoE values of cereal-based mixtures can be comparable with this study, including dietary exposure to Pb in China, where the MOE risk from dietary Pb averaged 1.57 and ranged from 0.13 to 6.18 [77]. It should be critically noted that this study dealt with the total daily intake of Pb, not just one serving of a selected food.

### 3.5. Results of the Evaluation of the Metal Pollution Index

The metal pollution index (MPI) is one of the basic indices which are evaluated as part of the human health risk assessment. Plant organisms predominantly absorb heavy metals from the soil; meanwhile, as industrialisation and agricultural modernisation levels rise, the risk of polluted farmland increases [78]. Being a consequential part of the human diet, foods require regular supervision to assess the level of various environmental xenobiotics, particularly heavy metals. In addition to water, the MPI index is now starting to be evaluated for food commodities such as fruits and vegetables [36,78,79], where the MPI reach values ten or more times higher than, for example, for cereals or noncereal flours [59,66]. Heavy metals, such as Zn and Cu, when present in trace amounts, are useful as micronutrients for the growth of humans; however, some toxic metals, such as Pb, Hg, and Cd, are detrimental to human health even at trace levels, particularly in pregnant women [78].

**Figure 7:**
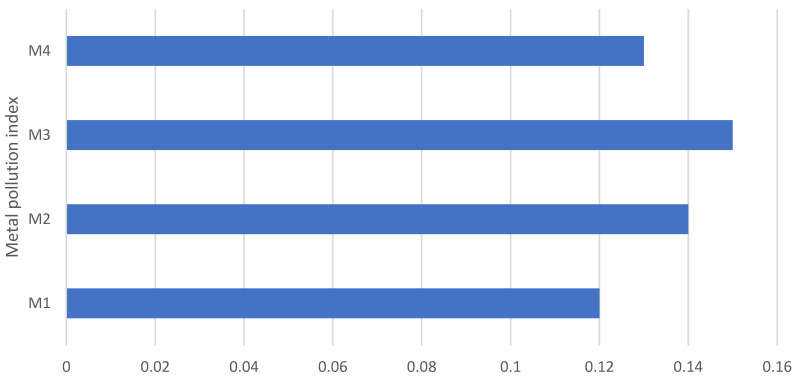
Metal pollution index of cereal-based mixtures.

Figure 7 represents the metal pollution index of the individual cereal-based mixtures examined, where the MPI value ranged from 0.12 to 0.15. In our study, heavy metals such as As, Pb, Hg, Fe, Cu, Zn, Ni, Cd, Co, Se, Ag, Tl, Cr, and Al were mentioned to calculate the MPI. A higher value of MPI signifies a higher concentration of heavy metal pollution; additionally, if the MPI values exceed 1.0, there is concern for potential health effects [78,79]. Our results are comparable to a study devoted to the accumulation of heavy metals in fruits and vegetables, where MPI values were in the range from 0.02 to 0.13, with the exception that only elements such as Pb, Cd, and Hg were included in the study [78]. Nevertheless, there are also studies that report higher MPI values for fruits and vegetables, in the range from 0.12 to 2.99, when Pb, Cd, As, Hg, and Cu were included in the calculation [79]. In the context of cereals as components of mixtures, non-traditional wheat grains and flakes had the highest MPI values of 0.033 and 0.024, respectively [59].

## 4. Conclusions

This study serves data on the content of essential and toxic elements in patented cereal-based mixtures containing non-traditional cereals, fruits, nuts, seeds, and edible flowers. Additionally, it estimates values of dietary intakes and exposures of essential and toxic trace elements and contributions to the appropriate reference values for adults of adults weighing 60, 80, and 100 kg when consuming a 50 g portion of the mixture as a breakfast. As a risk assessment value, the metal pollution index was evaluated.

Essential minerals and trace elements, such as K (up to 4150 µg/g), P, Mg, Ca, Na, Fe, Zn, Mn, and Cu (up to 22.8 µg/g), were found to be the most abundant elements in cereal-based mixtures. Concerning toxic trace elements, they were evaluated in the following order: Al (up to 12.1 µg/g) > Ni (up to 434 ng/g) > As > Ag > Pb > Hg > Cd > Sn (up to 1.12 ng/g); furthermore, Pb and Cd concentrations were measured in lower amounts than set by Commission Regulation (EU) 2023/915 on maximum levels for certain contaminants in food and repealing Regulation (EC) No 1881/2006. Taking into account ingestion of 50 g of cereal-based mixture per day for all age categories of women and men, the highest contributions to the AI value of Mn were obtained in the values reached 166 and 130%, respectively, while lower contribution values were recorded for the category of pregnant (150%) and lactating women (115%). In the case of consuming 50 g of mixtures, a consumer weighing 60 kg is most exposed to Al exposure (10.1 µg/kg bw/day), followed by exposure to Ni (362 ng/kg bw/day), As and Pb, Ag, Hg, Cd, and Sn (0.93 ng/kg bw/day). The highest contribution of non-traditional cereal-based mixtures to the toxic reference value was evaluated for As, which did not exceed 9% for an adult weighing 60 kg; furthermore, all remaining contributions of mixtures to the toxic dietary exposure values for Ag, Ni, Cd, and Al are less than 4%. In terms of Pb, the exposure values for developmental neurotoxicity, nephrotoxicity, and cardiovascular effects were considered safe. When the MPI value of the mixtures ranges from 0.12 to 0.15, there is no concern for potential detrimental health effects.

Based on our results and with respect to EU legislation, cereal-based mixtures with edible flowers could be considered and labelled a significant source of Mn, Cu, Zn, Fe, and P because they reached a minimum of 15% of the reference value for nutrients, in addition, labels such as low sodium/salt or very low sodium/salt can be applied because the mixtures contain no more than 0.12 g and 0.04 g of Na, respectively, per 100 g of product. Thus, new patented nutraceutical mixtures could help reduce the deficiency of the essential elements mentioned above in the adult population, pregnant and lactating women. Therefore, non-traditional cereal-based mixtures are suitable for use in the food industry as a potential source of essential minerals and trace elements for the adult human diet, including pregnant and lactating women, although bioaccessible and bioavailability studies should not be neglected in the future.

## 5. Patents

Patent No. 306520, 2017. Mlček, J.; Sumczynski, D. Nutraceutical food mixture. Industrial Property Office of the Czech Republic, Prague, Czech Republic.

## Figures and Tables

**Table 1:** Composition of raw materials in cereal-based mixtures.

Mixtures Containing Gluten				Gluten-Free Mixtures			
M1	g	M2	g	M3	g	M4	g
oat flakes	20	oat flakes	20	red rice flakes	20	red rice flakes	20
red wheat flakes	20	rye flakes	20	white teff flakes	20	black rice flakes	20
rye flakes	20	kamut flakes	20	black quinoa flakes	20	white quinoa flakes	20
hemp	2	hemp	2	hemp	2	hemp	2
almond	8	almond	8	almond	8	almond	8
hibiscus	2.5	rose	2	red cornflower	1.5	rose	1
lavender	0.5	mallow	1	blue cornflower	1.5	blue cornflower	2
raspberries	7	barberries	6	barberries	7	raspberries	7
barberries	6	apples	8	blueberries	7	barberries	6
apples	7	blueberries	8	cherries	7	apples	7
blueberries	7	strawberries	5	goldenberries	6	blueberries	7

**Table 2:** Minerals and trace elements assessed in cereal-based mixtures.

Analyte	M1	M2	M3	M4
(µg/g)				
^23^Na	130 ± 2 ^a^	137 ± 1 ^b^	160 ±8 ^c^	156 ± 7 ^d^
^24^Mg	1990 ± 20 ^a^	2530 ± 25 ^b^	2290 ± 20 ^b^	2310 ± 20 ^c^
^27^Al	10.8 ± 0.1 ^a^	12.1 ± 0.1 ^b^	5.41 ± 0.10 ^c^	7.72 ± 0.20 ^d^
^31^P	3850 ± 20 ^a^	3950 ± 30 ^b^	4070 ± 20 ^c^	3970 ± 20 ^d^
^39^K	2970 ± 20 ^a^	2970 ± 20 ^a^	4150 ± 30 ^b^	4050 ± 30 ^c^
^40^Ca	1640 ± 20 ^a^	2420 ± 20 ^b^	2380 ± 20 ^c^	2340 ± 20 ^d^
^55^Mn	17.4 ± 0.8 ^a^	17.8 ± 0.7 ^a^	19.1 ± 1.2 ^b^	59.7 ± 2.1 ^c^
^57^Fe	111 ± 3 ^a^	117 ± 2 ^b^	96.4 ± 2.7 ^c^	87.5 ± 1.4 ^d^
^63^Cu	7.04 ± 0.20 ^a^	6.21 ± 0.20 ^b^	22.8 ± 0.8 ^c^	6.21 ± 0.20 ^b^
^66^Zn	24.7 ± 0.7 ^a^	66.9 ± 1.7 ^b^	37.7 ± 1.1 ^c^	21.5 ± 0.5 ^d^
^137^Ba	3.61 ± 0.10 ^a^	4.40 ± 0.10 ^b^	3.84 ± 0.10 ^c^	4.20 ± 0.10 ^d^
(ng/g)				
^7^Li	15.3 ± 0.3 ^a^	19.3 ± 0.8 ^b^	24.8 ± 0.8 ^c^	24.4 ± 0.7 ^d^
^9^Be	12.3 ± 0.4 ^a^	11.6 ± 0.1 ^b^	3.84 ± 0.10 ^c^	6.82 ± 0.10 ^c^
^48^Ti	451 ± 8 ^a^	330 ± 9 ^b^	460 ± 10 ^c^	421 ± 8 ^d^
^52^Cr	2.11 ± 0.10 ^a^	8.22 ± 0.20 ^b^	4.82 ± 0.02 ^c^	5.61 ± 0.01 ^d^
^59^Co	32.9 ± 1.2 ^a^	26.5 ± 0.8 ^b^	31.8 ± 1.1 ^c^	31.4 ± 0.8 ^c^
^60^Ni	434 ± 5 ^a^	375 ± 3 ^b^	367 ± 5 ^c^	206 ± 2 ^d^
^75^As	28.6 ± 0.7 ^a^	24.1 ± 1.1 ^b^	14.2 ± 0.5 ^c^	31.5 ± 1.2 ^d^
^77^Se	7.81 ± 0.10 ^a^	9.60 ± 0.10 ^b^	16.3 ± 0.3 ^c^	17.6 ± 0.3 ^d^
^88^Sr	258 ± 3 ^a^	480 ± 5 ^b^	389 ± 2 ^c^	288 ± 3 ^d^
^107^Ag	15.4 ± 0.6 ^a^	12.1 ± 0.4 ^b^	31.4 ± 0.8 ^c^	14.2 ± 0.3 ^d^
^111^Cd	12.0 ± 0.1 ^a^	12.4 ± 0.1 ^a^	15.5 ± 0.10 ^b^	11.1 ± 0.10 ^c^
^118^Sn	1.12 ± 0.01 ^a^	0.64 ± 0.04 ^b^	0.52 ± 0.01 ^c^	0.84 ± 0.05 ^d^
^133^Cs	25.6 ± 0.2 ^a^	23.2 ± 0.5 ^b^	38.9 ± 1.1 ^c^	22.8 ± 0.4 ^b^
^140^Ce	16.3 ± 0.3 ^a^	18.4 ± 0.3 ^b^	44.6 ± 1.4 ^c^	43.8 ± 1.5 ^d^
^165^Ho	0.80 ± 0.01 ^a^	2.12 ± 0.02 ^b^	1.00 ± 0.01 ^c^	0.95 ± 0.01 ^c^
^181^Ta	<0.01	<0.01	<0.01	<0.01
^202^Hg	15.1 ± 0.1 ^a^	14.3 ± 0.1 ^b^	17.6 ± 0.5 ^c^	14.7 ± 0.1 ^b^
^205^Tl	1.80 ± 0.01 ^a^	2.22 ± 0.01 ^b^	1.22 ± 0.01 ^c^	2.20 ± 0.01 ^d^
^208^Pb	12.6 ± 0.2 ^a^	14.1 ± 0.4 ^b^	31.6 ± 0.4 ^c^	28.4 ± 0.4 ^d^
^238^U	<0.01	<0.01	<0.01	<0.01

The results are presented on a dry weight basis as means ± SD, *n* = 9 (the mean of nine measurements). Means within a line with at least one identical superscript do not differ significantly (*p* ≥ 0.05), means with various superscripts show a significant difference (*p *< 0.05). LOQ: Ta, U (0.01 ng/g).

**Table 3:** The results of exposures by body weight per day (DE_bw_) to toxic trace elements.

**Analyte**	**DE_60_ (µg/kg bw/day)**	**DE_80_ (µg/kg bw/day)**	**DE_100_ (µg/kg bw/day)**
Al	10.1	7.56	6.05
	**DE_60_ (ng/kg bw/day)**	**DE_80_ (ng/kg bw/day)**	**DE_100_ (ng/kg bw/day)**
Sn	0.93	0.70	0.56
Hg	14.7	11.0	8.80
Cd	12.9	9.69	7.75
Ni	362	271	217
As	26.3	19.7	15.8
Ag	26.2	19.6	15.7
Pb	26.3	19.8	15.8

## Data Availability

Data are contained within the article/Supplementary Materials.

## Supplementary Materials

The following supporting information can be downloaded at: https://www.mdpi.com/article/10.3390/nu17172848/s1. Table S1: RDA, AI*, PTWI, PTMI*, TDI, and RfD reference values defined for individual life stage groups or based on body weight.
