# Supplementary material for: Dietary Intakes and Exposures to Minerals and Trace Elements from Cereal-Based Mixtures: Potential Health Benefits and Risks for Adults
Source: Nutrients. 2025 Aug 31;17(17):2848. doi: 10.3390/nu17172848 (PMC12430257; doi:10.3390/nu17172848)
Supplement: Supplementary file 1 [file nutrients-17-02848-s001.zip › nutrients-3825037-supplementary.pdf]

Table S1. RDA, AI*, PTWI, PTMI*, TDI, and RfD reference values defined for individual life stage groups or based on the body weight.

**Essential minerals and trace elements**												
**Life stage group**	**Ca mg/day**	**Cr* μg/day**	**Cu μg/day**	**Fe mg/day**	**Mg mg/day**	**Mn* mg/day**	**Mo μg/day**	**P mg/day**	**Se μg/day**	**Zn mg/day**	**K* mg/day**	**Na* mg/day**
Men aged 19–30	1000	35*	900	8	400	2.3*	45	700	55	11	4700*	1500*
Women aged 19–30	1000	25*	900	18	310	1.8*	45	700	55	8	4700*	1500*
Pregnant women aged 19–30	1000	30*	1000	27	350	2.0*	50	700	60	11	4700*	1500*
Lactating women 19–30	1000	45*	1300	9	310	2.6*	50	700	70	12	5100*	1500*
Men aged 31–50	1000	35*	900	8	420	2.3*	45	700	55	11	4700*	1500*
Women aged 31–50	1000	25*	900	18	320	1.8*	45	700	55	8	4700*	1500*
Men aged 51–70	1000	30*	900	8	420	2.3*	45	700	55	11	4700	1300*
Women aged 51–70	1200	20*	900	8	320	1.8*	45	700	55	8	4700*	1300*

Men aged > 70	1200	30*	900	8	420	2.3*	45	700	55	11	4700*	1200*
Women aged > 70	1200	20*	900	8	320	1.8*	45	700	55	8	4700*	1200*
**Toxic trace elements**												
**μg/kg bw**	**Al**	**Sn**	**Hg**	**Cd***	**Ni**	**As**	Ag					
PTWI	2000	14000	4									
PTMI*				25*								
TDI					13	0.3						
RfD							5					

RDA – recommended dietary allowances, AI* – adequate intake (in case that the RDA value is not defined), PTWI – provisional tolerable weekly intake, PTMI* – provisional tolerable monthly intake, TDI – tolerable daily intake, RfD – reference dose, bw – body weight.
